# The Determination of Food Dyes in Vitamins by RP-HPLC

**DOI:** 10.3390/molecules21101368

**Published:** 2016-10-17

**Authors:** Monika Šuleková, Alexander Hudák, Miroslava Smrčová

**Affiliations:** Department of Chemistry, Biochemistry and Biophysics, Institute of Pharmaceutical Chemistry, The University of Veterinary Medicine and Pharmacy in Košice, Košice 04181, Slovak; alexander.hudak@uvlf.sk (A.H.); miroslava.smrcova@uvlf.sk (M.S.)

**Keywords:** food additive, synthetic dye, HPLC-DAD, vitamin

## Abstract

Reversed-phase high performance liquid chromatography (RP-HPLC) for the determination of five synthetic food dyes (Quinoline Yellow E104, Sunset Yellow E110, Ponceau 4R E124, Tartrazine E102 and Carmine E120) in vitamins was used. The dyes were analyzed within 10 min using a column with stationary phase C 18 (250 mm × 4.6 mm, 5 μm) at 40 °C with isocratic elution, and the mobile phase contained acetonitrile and a mixture of CH_3_COONa:CH_3_OH (85:15, *v*/*v*) in a ratio of 10:90 (*v*/*v*) for yellow-colored capsules and 20:80 (*v*/*v*) for red-colored capsules, respectively. A diode-array detector was used to monitor the dyes between 190 and 800 nm. It was established that the analyzed samples contained synthetic dyes in a concentration range from 79.5 ± 0.01 μg/capsule of Ponceau 4R, E124 to 524 ± 0.01 μg/capsule of Tartrazine, E102. The obtained results were compared with existing acceptable daily intakes (ADIs) for individual dyes. This paper provides information about the content of dyes in samples of vitamins. This information is not generally available to consumers.

## 1. Introduction

Vitamins constitute a significant group of essential substances [1,2]. They are ordinarily ingested by the consumption of fruit and vegetables. In the case of different health problems, fruit and vegetables are replaced by a variety of pharmaceutical products. There are supplements containing one vitamin or multivitamin preparations at disposal. These products often comprise varied additives in order to modify taste, visual appearance, or shape of the product. The appearance of vitamin preparations is often modified by the addition of different dyes, thereby becoming more attractive for consumers [3,4]. Coloring agents have a unique status as pharmaceutical excipients, and most regulatory agencies hold lists of colors that may be used in medicinal products. Natural or synthetic food dyes are one of the most widely used groups of excipients in the pharmaceutical industry. Dyes are added to foodstuffs, nutritional supplements, and drugs for commercial, psychological, and practical reasons. For coloring various products, the water-soluble substances of natural origin are used, or are prepared synthetically. Natural dyes are often unstable and easily subject to degradation by the effects of light, heat, or changing pH during the process of manufacture or storage, failing to serve their purpose. Therefore, natural dyes have been partially or completely replaced by synthetic counterparts, which have more advantages such as higher stability and a lower price [3,5,6].

The use of synthetic and natural dyes in foodstuffs and pharmaceuticals is strictly controlled by legislation and harmonized across the European Union. The compact list of coloring substances authorized for use in foodstuffs was determined in the European Parliament and Council Directive 94/36/EC about dyes for use in foodstuffs [7]. In this directive, the specific purity criteria concerning dyes for use in foodstuffs was established. The directive was clarified to the adoption of Regulation (EC) No. 1333/2008 of the European Parliament and Council on food additives and Directive 2009/35/EC of the European Parliament and Council on coloring substances that may be added to medicinal products [8]. Practical experience has shown that, in terms of health, there is no reason to disallow the use of foodstuff dyes in pharmaceutical products as well. There is a legal requirement for governments to monitor the consumption of all food additives in the European Union to ensure that acceptable daily intake values (ADI) are not exceeded, especially by young children [9].

Most synthetic dyes possess azo groups and aromatic rings in their chemical structure. Some of them, especially when eaten in large amounts, pose a potential risk to human health [10,11]. They can trigger undesirable effects, which can cause the appearance of allergies, asthma, and attention-deficit hyperactivity disorder (ADHD) [12,13,14,15]. For this reason, safety data, such as acceptable daily intake, based on toxicological studies on experimental animals and human clinical studies have been determined and evaluated by the Food and Agricultural Organisation and World Health Organisation [16]. A study by McCann et al. from 2007 concluded that exposure to a mixture including Tartrazine and Ponceau 4R resulted in increased hyperactivity in 3-year-old and 8–9-year-old children [17].

Sunset Yellow FCF (E110), Tartrazine (E102), and Ponceau 4R (E124) are azo dyes authorized as a food additive in the EU and have been previously evaluated by the Joint FAO/WHO Expert Committee on Food Additives and the Scientific Committee for Food. Quinoline Yellow (E104) is a quinophthalone dye, and Cochineal, Carminic acid, and Carmines (E120) are red anthraquinone dyes also allowed to be used as a food additive in the EU. Carmines and Carminic acid are defined, according to Commission Regulation (EU) No 231/2012, as being “obtained from aqueous, aqueous alcoholic or alcoholic extracts from Cochineal, which consist of the dried bodies of the female insect Dactylopius coccus Costa”. Carmines and Carminic acid are described as red to dark red friable solid or powder, while cochineal extract is generally a dark red liquid, but can also be dried as a powder [18].

Currently, there is sufficient attention given to the determination of dye content in food. In the case of pharmaceutical products, the situation is reversed, particularly in regard to preparations that are over-the-counter. This is the reason for the control of food dye content. This covers various tablets, capsules, syrups, and multivitamin supplements, but also some drugs that are attracted to children due to their color. The presence of dyes in these products is usually declared, but their content per tablet or capsule weight is rarely reported.

Many analytical methods have been developed for the identification and determination of food dyes, such as adsorptive voltammetry [19], differential pulse polarography [20], thin layer chromatography (TLC) [21], and spectrophotometry [22]. Ion-pair liquid chromatography [23] and reversed-phase high performance liquid chromatography (RP-HPLC) [6,24,25,26,27,28,29] are still the most preferred methods.

The aim of the current work was the identification and determination of food dyes by RP-HPLC with isocratic elution in a set of ten vitamin samples (vitamin E, vitamin C, vitamin B, and vitamin AD) from different producers. The used methodology for the determination of dyes was developed by Regional Public Health Authority in Kosice (Slovak).

## 2. Results and Discussion

The presence of the dyes in pharmaceutical products was evaluated in the samples of various vitamins. Due to the free availability of these products, it is not possible to control their consumption, which is particularly increasing in the winter season. Ten samples of vitamins were obtained from the pharmacy in order to analyze. These were orange-colored capsules of Vitamin C (Celaskon long effect, Zentiva, Prague, Czech Republic), white tablets of Vitamin C 1000 (Green Swan Pharmaceuticals, Prague, Czech Republic ), red gelatine capsules of Vitamin E (Zentiva, Generica, Noventis), colorless gelatine capsules of Vitamin E (Dr. Max Jamieson), yellow gelatine capsules of Vitamin AD (Slovakofarma, Hlohovec, Slovakia), and yellow tablets of B-complex vitamins (Zentiva).

Analyses of individual samples were based on the information of their composition, which is declared on the package leaflet by the producers. Companies Dr. Max, Jamieson, and Green Swan reported no use of the dyes in their products. Ponceau 4R is mentioned between additives on the package leaflet of Vitamin E (Zentiva). Other producers such as Generica and Noventis apply the dye Carmine for coloring the Vitamin E capsules. Producer Zentiva declared Sunset Yellow to be used in the capsules of Celaskon long effect and Quinoline Yellow, Sunset Yellow, and Ponceau 4R in B-complex tablets.

**Figure 1:**
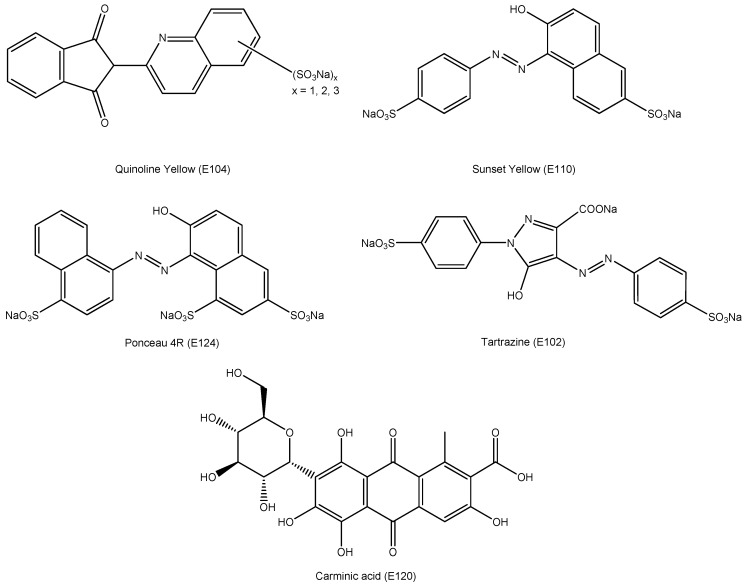
Chemical structures of the studied dyes.

Analyzed food dyes (Figure 1) Quinoline Yellow (E104), Sunset Yellow FCF (E110), Ponceau 4R (Cochineal red, E124), Tartrazine (E102), and Carmine (Cochineal, Carminic acid, E120) are extensively used in the pharmaceutical industry.

**Figure 2:**
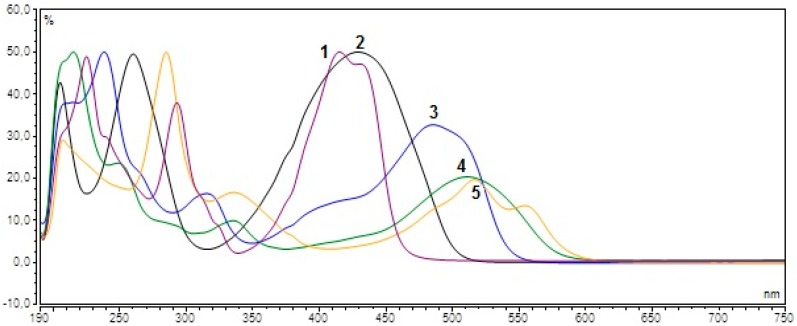
UV/VIS spectra of coloring standards. Peaks identity: 1—Quinoline Yellow (E104); 2—Tartrazine (E102); 3—Sunset Yellow (E110); 4—Ponceau 4R (E124); 5—Carmine (E120).

Presence of the dyes in the vitamin samples was identified by measuring UV/VIS spectra of all the sample solutions and standard solutions of dyes Quinoline Yellow (20.0 μg·mL^−1^), Sunset Yellow (5.0 μg·mL^−1^), Ponceau 4R (5.0 μg·mL^−1^), Tartrazine (40.0 μg·mL^−1^) and Carmine (15.0 μg·mL^−1^) which structures are shown in Figure 1. The measurements were performed regardless of whether the producer declared the presence of the colorant or not. Comparing UV/VIS spectra of the samples with spectra of the standard solutions, the presence of the following dyes was revealed in some samples: Quinoline Yellow, Sunset Yellow, Ponceau 4R, Tartrazine, and Carmine. The UV/VIS spectra records of dyes are shown in Figure 2. The optimal absorption wavelength for each colorant was determined by measuring the standard solutions. Based on these measurements, the following wavelengths for the quantitative analysis were used: Quinoline Yellow (425 nm), Sunset Yellow (480 nm), Ponceau 4R (510 nm), Tartrazine (425 nm), and Carmine (520 nm).

**Figure 3:**
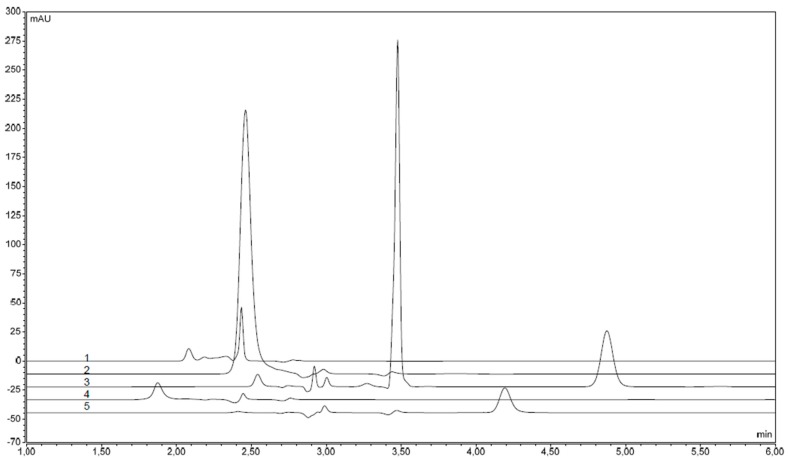
Chromatograms of coloring standards: 1—Ponceau 4R (E124); 2—Tartrazine (E102); 3—Quinoline Yellow (E104); 4—Carmine (E120); 5—Sunset Yellow (E110).

The reversed-phase high performance liquid chromatography method with the diode array detector was used for the quantitative analysis of the dyes. Corresponding chromatograms are illustrated in Figure 3. In pharmaceutical products, food dyes were identified by comparison of their retention times with those of standards. Retention times of dyes in the studied samples under the given experimental conditions are listed in Table 1. All food dyes were eluted in less than 5 min. To evaluate the obtained results, the calibration of signals was performed by measuring the standard solutions. The equations of calibration functions were obtained by the software Chromeleon Chromatography Data System, Version 7.2 (Thermo Fisher Scientific, Waltham, MA, USA) using peak areas. The linearity of the calibration function was calculated by the least squares method. Linear equations of the reference solutions, correlation coefficients, limit of detection (LOD), limit of quantification (LOQ), and residual standard deviations for each determined colorant are presented in Table 2. LOD and LOQ values (by Habaux and Vos) were assessed using the above-mentioned software. In each case, three repeated measurements were performed in three parallel determinations.

The presence of dyes was confirmed in six of ten analyzed samples. There was no signal of dyes detected in the following products: Vitamin C 1000 (Green Swan), Vitamin E (Dr. Max and Jamieson), and B-complex (Zentiva). Potentially present content in dyes were below the LOD value.

The results of analyzed samples in which manufactures declare the presence of dyes are shown in Table 2. In the mentioned samples, the following synthetic dyes were determined: Sunset Yellow, Ponceau 4R, and Tartrazine. Carmine was also quantified. In the products in which producers stated no use of dye addition, there was no dye detected via HPLC analysis. These samples are not included in this table.

The use of synthetic and natural dyes in foods and dietary supplements is governed by the relevant rules, which are regularly reviewed. The Joint FAO/WHO Expert Committee on Food Additives (JECFA) in 1983 and the EU Scientific Committee for Food (SCF) in 1984 established an ADI of 0–4.0 mg/kg bw/day for Ponceau 4R and of 0–7.5 mg/kg bw/day for Tartrazine. The European Food Safety Authority issued an Opinion in 2009 related to the re-evaluation on the safety of Quinoline Yellow, Sunset Yellow FCF, and Ponceau 4R as a food additive. In that Opinion, the Authority recommended decreasing the ADI value of Quinoline Yellow from 10.0 mg/kg bw/day to 0.5 mg/kg bw/day, the ADI of Sunset Yellow FCF from 2.5 to 1 mg/kg bw/day, and the ADI of Ponceau 4R from 4.0 mg/kg bw/day to 0.7 mg/kg bw/day. The acceptable daily intake value of carmine, which is formulated by the Joint FAO/WHO Expert Committee on Food Additives, is 5.0 mg·kg^−1^ based on weight [30,31].

The content of dyes calculated per each type of capsule can be seen in Table 2. To compare the obtained results with ADI values of the individual dyes mentioned above, consider the following example of 70 kg/person. To achieve an ADI upper limit of Ponceau 4R (0.7 mg/kg bw/day), this person would have to consume almost 62 capsules of vitamin E (Zentiva) in one day. In case of Carmine (5.0 mg/kg bw/day) and Tartrazine (7.5 mg/kg bw/day), this would represent 707 capsules of vitamin E (Generica) or 1112 capsules of vitamin E (Noventis) and 1002 capsules of vitamin AD, respectively. Various forms of vitamin C belong to the most popular vitamin products. Celaskon long effect (Zentiva) is colored by Sunset Yellow with an ADI upper value 1.0 mg/kg bw/day, which represents 440 capsules consumed by one person in one day.

Based on the obtained results it can be stated that some manufacturers do not use any additives such as dyes; some are focused on using natural coloring substances or their synthetic counterparts. The content of synthetic dyes that corresponds to one capsule or tablet of analyzed sample was too low in consideration to valid ADI values. There is no precondition for the occurrence of health problems when consumption is appropriate and controlled.

## 3. Materials and Methods

### 3.1. Chemicals and Solutions

Gradient-grade methanol for HPLC (Sigma-Aldrich, St. Louis, MO, USA) and Acetonitrile for LC-MS (Merck, Prague, Czech Republic) was used for analysis. Solid dye standard of Quinoline Yellow, E104, Ponceau 4R, E124, and Tartrazine, E102, were obtained from the Institute for Engineering of Polymer Materials and Dyes, Department of Dyes and Organic Products, in Zgierz (Poland). The standard of Sunset Yellow FCF and the standard of Carmine were obtained from Sigma-Aldrich (USA). All standards were of HPLC grade. Analytical grade sodium acetate trihydrate was supplied from LachNer (Neratovice, Czech Republic). Ultra-pure water (Purelab Flex with purification pack: LC208) was used for the preparation of all solutions. A working solution was prepared by dissolving the appropriate amounts of powder of each color to obtain a concentration of 0.5 mg·mL^−1^. Calibration standards of dye were prepared by the dilution of aliquots of the stock working solutions. The concentration range for standard solutions was 5–60 μg·mL^−1^ of Quinoline Yellow, 0.5–45 μg·mL^−1^ of Sunset Yellow, 1–10 μg·mL^−1^ of Ponceau 4R, 5–60 μg·mL^−1^ of Tartrazine and 2.5–15 μg·mL^−1^ of Carmine. All solutions were stored in dark flasks at 5 °C.

### 3.2. Sample Preparation

All tested samples of vitamins E, vitamins C, vitamin AD, and vitamin B (produced by different manufacturers—Zentiva, Noventis, Dr. Max, Generica, Slovakofarma, GreenSwan pharmaceuticals, and Jamieson) were obtained from a local pharmacy. The gelatine capsules and tablets of vitamins were accurately weighed and dissolved in water via heating. The sample solutions were placed in the ultrasonic bath (Sonorex Digitec, Bandelin, Germany) for 15 min to complete the colorant extraction, followed by centrifugation (B. Braun Sigma 2 K15, Melsungen, Germany) at 19,000 rpm for 15 min. These solutions were filtered through a folder paper filter (0.4 µm) to achieve more efficient filtration. The filtrate was collected in a volumetric flask of 10 mL. All solutions were injected after filtering. One milliliter of supernatant liquid was then transferred into an HPLC autosampler vial for an injection of 20 μL onto the column. There three repeated measurements were performed in three parallel determinations.

### 3.3. Instrumentation and HPLC Analysis

The pharmaceutical samples of vitamins were analyzed using a high-performance liquid chromatography (HPLC). The HPLC system Dionex UltiMate 3000 RS (Thermo Fisher Scientific, Waltham, Germany) consisted of a quaternary pump, degasser, automated injector, column oven, and diode array detector (DAD). The DAD detector was set to collect signals within the spectral range of 190–800 nm. Chromatographic separation was performed on the chromatographic column (250 mm × 4.6 mm) Polaris C18-A at a particle size of 5 μm (Varian, Santa Clara, CA, USA). The injection volume was 20 μL. During the chromatographic separation, the mobile phase was kept isocratic, at a flow rate of 1.2 mL·min^−1^ for red-colored capsules and at a flow rate of 1.0 mL·min^−1^ for yellow-colored capsules. The mobile phase system consisted of two components: A (CH_3_CN) and B (CH_3_COONa:CH_3_OH = 85:15, *v*/*v*) in a ratio of 20:80, *v*/*v* for red colored capsules, and in a ratio of 10:90, *v*/*v* for yellow-colored capsules, respectively. The column was kept at 40 °C in a column oven. Analyses were performed with Chromeleon Chromatography Data System, Version 7.2 (Thermo Fisher Scientific, Waltham, Germany) for collecting and processing data. Each analysis was performed in three replicates.

## 4. Conclusions

The content of dyes (Quinoline Yellow, Sunset Yellow, Ponceau 4R, Tartrazine, and Carmine) was determined by RP-HPLC with isocratic elution and the DAD detector in real samples of various vitamins. It was experimentally confirmed that dyes are added to vitamin preparations in small amounts. The values of dye content in all of analyzed pharmaceutical samples did not exceed the maximum permitted level. From this perspective, in some cases, one would have to consume a few hundred vitamin tablets to exceed the ADI value. Considerable attention is paid to the use of synthetic dyes in foodstuffs. In spite of the fact that dyes are used in the manufacture process of pharmaceuticals, there is often a lack of interest. Usually, producers do not declare the origin of dyes that have been used, nor do they declare the added content per tablet or capsule of the product.

## Figures and Tables

**Table 1:** Retention time of studied samples; the content of dyes in the vitamin samples.

Sample	Retention Time (min)	Concentration of Colorant (μg·mL^−1^)	Content of Colorant (μg/Capsule)
Vitamin E (Generica)	1.830	49.50 (E120)	495 ± 0.02
Vitamin E (Noventis)	1.870	31.48 (E120)	314.8 ± 0.02
Vitamin E (Zentiva)	2.420	7.95 (E124)	79.5 ± 0.01
Vitamin AD (Slovakofarma)	2.463	52.40 (E102)	524.0 ± 0.01
Celaskon long effect (Zentiva) body	4.337	4.00 (E110)	40.0 ± 0.01
Celaskon long effect (Zentiva) cap	4.340	11.90 (E110)	119.0 ± 0.01

**Table 2:** Chromatographic characteristics of the method for the high performance liquid chromatography (HPLC) analysis of dyes.

Colorant	Wavelength (nm)	Calibration Equation	LOD (μg·mL^−1^)	LOQ (μg·mL^−1^)	Correlation Coefficient (*n* = 3)	RSD (%)
Quinoline Yellow (E104)	425	*y* = 465.0856*x* − 0.1182	1.00	3.32	0.9994	0.464
Sunset Yellow (E110)	480	*y* = 562.5234*x* − 0.0315	0.40	1.33	0.9995	1.088
Ponceau 4R (E124)	510	*y* = 154.5389*x* + 0.2086	0.70	2.33	0.9991	1.784
Tartrazine (E102)	425	*y* = 580.9923*x* − 0.2535	3.72	12.38	0.9993	1.663
Carmine (E120)	520	*y* = 86.1634*x* − 0.0242	2.40	7.99	0.9989	2.421
